# Protective Effects of Fasudil Against Cisplatin-Induced Ototoxicity in Zebrafish: An In Vivo Study

**DOI:** 10.3390/ijms252413363

**Published:** 2024-12-13

**Authors:** Kang Hyeon Lim, Saemi Park, Eunjung Han, Hyun woo Baek, Kyungtae Hyun, Sumin Hong, Hwee-Jin Kim, Yunkyoung Lee, Yoon Chan Rah, June Choi

**Affiliations:** 1 Department of Otorhinolaryngology-Head and Neck Surgery, Korea University College of Medicine, Ansan Hospital, Ansan 15355, Republic of Korea; kingsonl@hanmail.net (K.H.L.); babydazzler@gmail.com (S.P.); hoj7843@korea.ac.kr (E.H.); bhw0810@naver.com (H.w.B.); vlalr@naver.com (K.H.); hongsumin0608@naver.com (S.H.); hweejin2@gmail.com (H.-J.K.); leeyk83@gmail.com (Y.L.); ycrah@naver.com (Y.C.R.); 2 Zebrafish Translational Medical Research Center, Korea University, Ansan 15355, Republic of Korea

**Keywords:** fasudil, ROCK inhibitor, zebrafish, ototoxicity, cisplatin, hair cell

## Abstract

While cisplatin is an effective anti-tumor treatment, it induces ototoxicity through mechanisms involving DNA damage, oxidative stress, and programmed cell death. Rho-associated coiled-coil-containing protein kinase (ROCK) is essential for numerous cellular processes, including apoptosis regulation. Studies have suggested that ROCK inhibitors could prevent apoptosis and promote regeneration. We aimed to investigate the protective effects of the ROCK inhibitor fasudil against cisplatin-induced ototoxicity in a zebrafish model. The zebrafish larvae were exposed to 1 mM cisplatin alone or 1 mM cisplatin co-administered with varying concentrations of fasudil for 4 h. The surviving hair cell counts, apoptosis, reactive oxygen species (ROS) levels, mitochondrial membrane potential (ΔΨm), caspase 3 activity, and autophagy activation were assessed. Rheotaxis behavior was also examined. Cisplatin reduced hair cell counts; increased apoptosis, ROS production, and ΔΨm loss; and activated caspase 3 and autophagy. Fasudil (100 and 500 µM) mitigated cisplatin-induced hair cell loss, reduced apoptosis, and inhibited caspase 3 and autophagy activation. Rheotaxis in zebrafish was preserved by the co-administration of fasudil with cisplatin. Cisplatin induces hair cell apoptosis in zebrafish, whereas fasudil is a promising protective agent against cisplatin-induced ototoxicity.

## 1. Introduction

Clinically, cisplatin possesses excellent efficacy in shrinking the size of solid or central nervous system tumors [1,2]. However, it is accompanied by adverse effects including nephrotoxicity, neurotoxicity, and ototoxicity, and the prevalence of hearing impairment is contingent upon the cumulative dose [3]. Cisplatin ototoxicity presents as irreversible, progressive, bilateral sensorineural hearing loss, and the mechanism of outer hair cell death in the organ of Corti is apoptosis [3,4]. The platinum–DNA complex, which forms after cisplatin enters the cell nucleus, induces DNA damage and oxidative stress, leading to mitochondrial dysfunction, endoplasmic reticulum stress, and the activation of autophagy, culminating in programmed cell death [3,5]. However, the precise mechanisms of hair cell damage and potential preventive therapies for cisplatin-induced ototoxicity are under investigation [5].

Numerous studies have demonstrated that rho-associated coiled-coil-containing protein kinase (ROCK) is essential for cell contraction, migration, apoptosis, survival, and proliferation [6,7,8,9]. ROCK can regulate apoptosis, and the Rho/ROCK signaling pathway is substantially involved in the regulation of cisplatin-induced apoptosis in human embryonic stem cells, neuroblastoma cells, and rodent models [7,10,11,12,13,14,15]. Studies have demonstrated the efficacy of ROCK inhibitors in preventing apoptosis and promoting the regeneration of neuronal synapses and axons by suppressing the Rho/ROCK pathway [16,17]. Additionally, a study showed that ROCK inhibitors can block neomycin-induced hair cell damage [18].

The lateral line of zebrafish (Danio rerio) consists of neuromasts, which are bundles of hair cells that detect stimuli such as surrounding flow, vibrations, and pressure waves [19]. The hair cells of zebrafish are structurally and functionally analogous to those located in the mammalian inner ear [20,21]. Therefore, zebrafish models have been broadly applied in research on hair cell function, hearing loss, and ototoxicity [19,21,22,23,24,25,26]. Studies have indirectly quantified the extent of hair cell damage in neuromasts by observing the behaviors regulated by these structures in zebrafish [27,28,29,30,31,32,33,34,35]. The loss of rheotaxis, a behavior in which zebrafish orient themselves to swim against the current by detecting surrounding flow, is notably correlated with the degree of hair cell damage in the lateral line [29,31,32,33,36,37,38,39,40].

Therefore, we aimed to examine the effect of Rho/ROCK pathway inhibition with the representative ROCK inhibitor fasudil (an isoquinoline sulfonamide derivative) on cisplatin-induced ototoxicity [6]. Our findings underscore the potential of fasudil as a therapeutic agent to prevent hearing loss in patients receiving cisplatin-based chemotherapy.

## 2. Results

### 2.1. Effect of Fasudil on Hair Cell Count in Neuromasts

**Figure 1:**
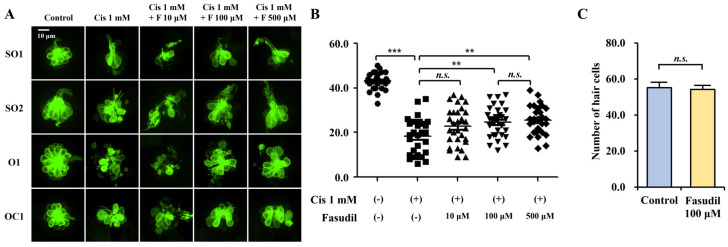
Effect of fasudil on the hair cells in zebrafish. (**A**) Cisplatin reduced the number of hair cells in neuromasts, while fasudil protected hair cells from cisplatin-induced ototoxicity, with increasing concentrations resulting in the salvage of a greater number of hair cells (×20; scale bar = 10 μm). (**B**) Fasudil protected against cisplatin-induced hair cell loss at concentrations of 100 μM and 500 μM (n = 30 per group; *** *p* < 0.001; ** *p* < 0.01). (**C**) The administration of 100 μM fasudil did not induce significant hair cell loss compared with the controls (n = 20 per group; *p* = 0.258). Abbreviations—*n.s.*: not significant.

To assess the alterations in the number of hair cells following the administration of cisplatin alone or in combination with various concentrations of fasudil, the hair cell counts in the neuromasts across four regions (SO1, SO2, O1, and OC1) were determined. The residual hair cell count was calculated as a percentage of that in the control group, which was treated with neither fasudil nor cisplatin (Table 1; Figure 1A,B). Cisplatin induced a significant depletion in the number of hair cells within the neuromasts, whereas fasudil conferred considerable protection against cisplatin-induced hair cell loss at concentrations of 100 μM and 500 μM. The number of hair cells was as follows: control group (n = 30): 43.1 ± 3.6; cisplatin 1 mM group (n = 30): 18.4 ± 7.9; fasudil 10 μM group (n = 30): 22.9 ± 8.2; fasudil 100 μM group (n = 30): 24.7 ± 6.7; fasudil 500 μM group (n = 30): 25.5 ± 6.2 cells (*p* < 0.001, ANOVA).

No significant difference was observed between the 100 and 500 μM concentrations of fasudil. Therefore, for subsequent experiments, we used a concentration of 100 μM to analyze the protective effect of fasudil against cisplatin-induced hair cell loss. In the cytotoxicity assay, no significant hair cell loss was observed in the 100 μM fasudil group (Figure 1C). The number of hair cells was as follows: control group (n = 20): 55.2 ± 2.9; fasudil 100 μM group: 54.3 ± 3.0 (n = 20) (*p* = 0.258, Mann–Whitney test).

### 2.2. Effect of Fasudil on Cellular Apoptosis

**Figure 2:**
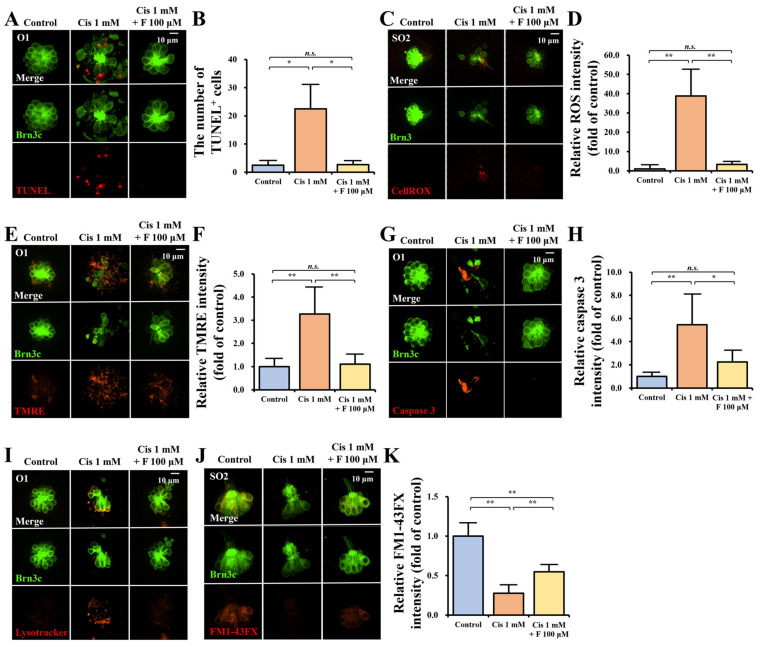
Protective effect of fasudil against cisplatin-induced apoptosis and hair cell viability. (**A**) Apoptotic cells were observed as red dots in the O1 neuromast of 5-dpf transgenic zebrafish after TUNEL staining under a fluorescent microscope (×20; scale bar = 10 μm). (**B**) The total number of TUNEL-positive cells in four neuromasts (SO1, SO2, O1, and OC1) was compared across the groups (* *p* < 0.05). (**C**) Red dots indicating ROS generation were observed in the SO2 neuromast after CellROX deep red staining under a fluorescent microscope. (×20; scale bar = 10 μm). (**D**) The average relative ROS intensity of the four neuromasts (SO1, SO2, O1, and OC1) was compared among groups, using the control group as reference (** *p* < 0.01). (**E**) Red dots indicating mitochondrial membrane potential loss were observed in the O1 neuromast with tetramethylrhodamine ethyl ester (TMRE) staining, under a fluorescent microscope (×20; scale bar = 10 μm). (**F**) The average relative TMRE intensity of the four neuromasts (SO1, SO2, O1, and OC1) was compared among the groups, with the control group as reference (** *p* < 0.01). (**G**) Red dots indicating caspase 3 activity were observed in the O1 neuromast following anti-Caspase 3 antibody staining under fluorescent microscopy (×20; scale bar = 10 μm). (**H**) The average relative caspase 3 labeling intensity of the four neuromasts (SO1, SO2, O1, and OC1) was compared among the groups, with the control group as reference (* *p* < 0.05, ** *p* < 0.01). (**I**) Red dots indicating autophagy activation were observed in the O1 neuromast after LysoTracker Red DND-99 staining under fluorescent microscopy (×20; scale bar = 10 μm). (**J**) Red dots indicating hair cell viability were observed in the SO2 neuromast after FM1-43FX staining (×20; scale bar = 10 μm). (**K**) The average relative FM1-43FX intensity of the four neuromasts (SO1, SO2, O1, and OC1) was compared among the groups, with the control group as reference (** *p* < 0.01). Abbreviations—*n.s.*: not significant; Cis: cisplatin; F: fasudil; dpf: days post-fertilization; TUNEL: terminal deoxynucleotidyl transferase (TdT)-mediated dUTP– biotin nick end labeling; ROS: reactive oxygen species; SO1 and SO2: supraorbital; O1: otic; OC1: occipital.

We conducted a quantitative assay of apoptosis induced by cisplatin in the neuromasts using TUNEL staining (Figure 2A,B). In the TUNEL assay, the group that was administered 1 mM cisplatin exhibited a notable rise in the count of TUNEL-positive hair cells in four neuromasts (SO1, SO2, O1, and OC1) compared with the control group; however, the group co-treated with 1 mM cisplatin and 100 μM fasudil showed a marked reduction in the TUNEL-positive hair cell number compared with the cisplatin-only group. Additionally, there was no significant difference in the count of TUNEL-positive hair cells between the control and cisplatin + fasudil groups. Thus, fasudil exerted a significant protective effect against cisplatin-induced apoptosis in zebrafish hair cells. The number of TUNEL-positive cells was as follows: control group (n = 4): 2.5 ± 1.7; cisplatin group (n = 6): 22.5 ± 8.5; and cisplatin + fasudil group (n = 3): 2.7 ± 1.2 (*p* = 0.010, Kruskal–Wallis test).

ROS production in zebrafish hair cells during cisplatin-induced apoptosis was confirmed via quantitative analysis with CellROX deep red staining (Figure 2C,D). The relative ROS intensity was significantly elevated in the cisplatin group compared to the control group, whereas the relative ROS intensity in the cisplatin + fasudil group was significantly lower than that in the cisplatin group. Additionally, no notable difference was detected in the relative ROS intensity between the control and cisplatin + fasudil groups. The relative ROS intensity (determined using the ROS intensity of the control group as reference) was as follows: control group (n = 6): 1.0 ± 1.1; cisplatin 1 mM group (n = 6): 38.8 ± 13.6; fasudil 100 μM group (n = 6): 3.3 ± 0.8 (*p* = 0.002, Kruskal–Wallis test).

Furthermore, quantitative analysis using TMRE was performed to confirm the loss of ΔΨm in zebrafish hair cells during cisplatin-induced apoptosis (Figure 2E,F). The relative TMRE intensity was significantly higher in the cisplatin group compared to the control group, whereas the relative labeling intensity in the cisplatin + fasudil group was significantly lower than that in the cisplatin group. Additionally, no significant difference was observed in the relative labeling intensity between the control and cisplatin + fasudil groups. The relative TMRE intensity based on the intensity of the control group was as follows: control group (n = 4): 1.0 ± 0.3; cisplatin group (n = 7): 3.3 ± 1.1; cisplatin + fasudil group (n = 7): 1.1 ± 0.4 (*p* = 0.02, Kruskal–Wallis test).

We also analyzed the activation of caspase 3 and autophagy during cisplatin-induced ototoxicity. Staining with the C8487 Anti-Caspase 3 antibody revealed an increase in caspase 3 activity in the cisplatin-only group compared to the control group, whereas co-treatment with cisplatin and fasudil resulted in a reduction in caspase 3 activity (Figure 2G,H). The relative caspase 3 labeling intensity was significantly higher in the cisplatin group compared to the control group, whereas the relative labeling intensity in the cisplatin + fasudil group was significantly lower than that in the cisplatin group. Additionally, no significant difference was observed in the relative labeling intensity between the control and cisplatin + fasudil groups. The caspase 3 labeling intensity relative to that of the control group was as follows: control group (n = 5): 1.0 ± 0.3; cisplatin group (n = 5): 5.5 ± 2.7; cisplatin + fasudil group (n = 5): 2.2 ± 1.0 (*p* = 0.05, Kruskal–Wallis test).

Additionally, staining with LysoTracker Red DND-99 revealed greater autophagy activation in the cisplatin-only group compared to the control group, whereas co-treatment with cisplatin and fasudil led to a decline in autophagy activation (Figure 2I). Therefore, we qualitatively confirmed that fasudil inhibits caspase 3 activation and autophagy activation in cisplatin-induced ototoxicity.

### 2.3. Analysis of Hair Cell Viability for Determining the Protective Effect of Fasudil Against Cisplatin

We used FM1-43FX staining to evaluate the functionality of the surviving hair cells in the four neuromasts (SO1, SO2, O1, and OC1) (Figure 2J,K). The mechanotransduction function of hair cells after FM1-43FX treatment was assessed based on the fluorescence intensity under microscopy. As shown in Figure 2J, the control SO2 neuromasts exhibited robust labeling intensity in hair cells, whereas neuromast hair cells treated with cisplatin displayed a weaker labeling intensity. However, co-treatment with cisplatin and fasudil preserved the labeling intensity. Compared with that in the control group, the FM1-43FX intensity was significantly lower (quantitatively) in the cisplatin-treated group. Although the groups treated with both cisplatin and fasudil also showed a decrease in relative labeling intensity relative to the control group, it was significantly preserved compared with the group treated with cisplatin alone (Figure 2K). The relative FM1-43FX intensity, based on the intensity of the control group was as follows: control group (n = 6): 1.0 ± 0.2; cisplatin group (n = 6): 0.27 ± 0.1; cisplatin + fasudil group (n = 6): 0.5 ± 0.1 (*p* = 0.001, Kruskal–Wallis test).

### 2.4. Effect of Fasudil on the Behavior of Transgenic Zebrafish Larvae Treated with Cisplatin

**Figure 3:**
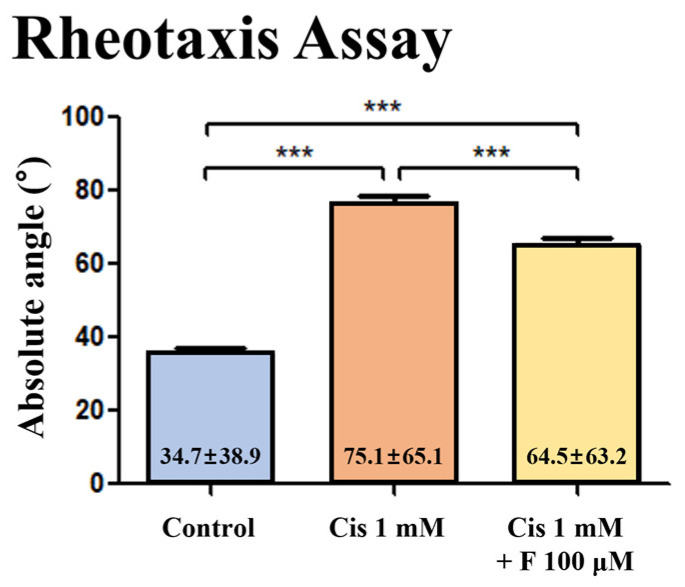
Effects of fasudil on the behavior of transgenic zebrafish treated with cisplatin. Compared with the control group, the group treated with 1 mM cisplatin alone exhibited a significant increase in the absolute angle of rheotaxis. The group treated with 1 mM cisplatin and 100 μM fasudil showed a significantly decreased absolute angle compared with the group treated with cisplatin alone (n = 30 per group; *** *p* < 0.001). The average absolute angle of the larvae is shown at the bottom of each bar graph.

To compare the degree of rheotaxis among all groups, the absolute angles formed by the larvae in still images captured every 2.5 s over 4 min (960 larval images; 4 min × 24 images/min × 10 larvae/image) were measured and compared. Compared with the control group, the group treated with 1 mM cisplatin alone exhibited a significantly greater absolute angle, indicating a significant loss of rheotaxis. Conversely, the group treated with both 1 mM cisplatin and 100 μM fasudil showed a significantly lower absolute angle compared with the group treated with cisplatin alone (Figure 3). The absolute angles were as follows: control group (n = 30): 34.7 ± 38.9°; cisplatin group (n = 30): 75.1 ± 65.1°; cisplatin + fasudil group (n = 30): 64.5 ± 63.2° (*p* < 0.001, ANOVA).

## 3. Discussion

In the current study, cisplatin treatment increased lateral line hair cell loss and altered rheotactic behavior in a transgenic zebrafish model. Cisplatin-induced cellular changes in zebrafish were characterized by increased ROS levels in the cytoplasm, decreased mitochondrial transmembrane potential, increased caspase-3 activity, the activation of autophagy, and enhanced apoptosis. These findings align with prior studies exploring the mechanism of zebrafish hair cell changes induced by cisplatin [28,41,42,43].

**Figure 4:**
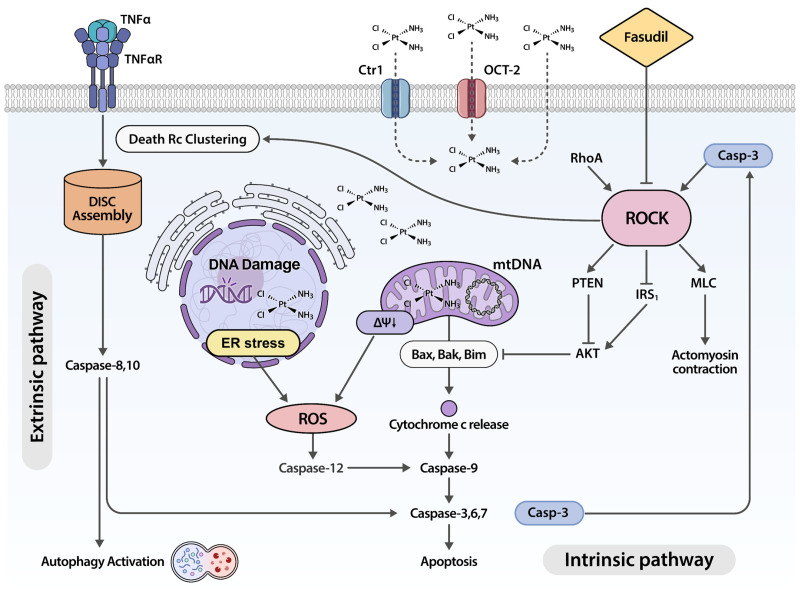
Hypothetical illustration of the role of ROCK in cisplatin-induced apoptosis. When cisplatin enters the cytoplasm of hair cells via CTR1 and OCT2 transporters or membrane diffusion, it binds to nuclear or mitochondrial DNA, triggering ROS generation through ER stress and the loss of mitochondrial transmembrane potential. Cytochrome C released due to ROS and mitochondrial damage activates caspases, promoting the intrinsic pathway of apoptosis. In this process, ROCK activated by caspase-3 inhibits AKT through the activation of PTEN and the inhibition of IRS1. The inhibition of AKT accelerates the release of cytochrome C by activating Bax, Bak, and Bim, thereby forming a positive feedback loop. Additionally, ROCK induces the extrinsic apoptosis pathway via the clustering of death receptors such as TNF-α, wherein the activation of caspase-8 leads to autophagy activation. In the apoptotic execution phase, ROCK also induces actomyosin contraction through the phosphorylation of MLC. Fasudil is thought to mitigate cisplatin-induced hair cell apoptosis by inhibiting ROCK. Abbreviations—CTR1: copper uptake protein 1; OCT2: organic cation transporter 2; ROS: reactive oxygen species; ER: endoplasmic reticulum; RhoA: Ras homolog family member A; ROCK: Rho-associated protein kinase; PTEN: phosphatase and tensin homolog; IRS1: insulin receptor substrate 1; Bax: bcl-2-like protein 4; Bak: Bcl-2 homologous antagonist/killer; TNF-α: tumor necrosis factor-α; DISC:, death-inducing signaling complex; MLC: myosin light chain.

While the exact mechanism underlying cisplatin-induced ototoxicity is not completely understood, numerous studies have suggested that ototoxicity arises from programmed cell death, such as apoptosis, necroptosis, autophagic cell death, ferroptosis, and pyroptosis of hair cells, which accompanies oxidative stress [1,2,3,5,44]. Oxidative imbalance occurs as a result of the overproduction of ROS following nuclear and mitochondrial DNA damage induced by cisplatin, and endoplasmic reticulum stress (Figure 4) [3,5]. The accumulation of ROS within hair cells disrupts the redox balance, eliciting the apoptotic pathway [2,5]. Prior research has demonstrated that cisplatin triggers apoptosis in hair cells via the induction of the intrinsic apoptotic pathway mediated by the mitochondria. This process is marked by an elevated Bax/Bcl-2 ratio, a reduction in ΔΨm, and the release of cytochrome C, which induces the activation of caspase-3 [1,3,5]. Moreover, research has shown that caspase-8, which is activated by the clustering of death receptors such as the tumor necrosis factor-α (TNF-α) receptor, a component of the extrinsic apoptotic pathway, is activated in cisplatin ototoxicity [43,45]. Other studies have reported that cisplatin treatment upregulates the expression of recognized autophagy markers, including Beclin-1, microtubule-associated protein light chain 3, and nucleotide-binding oligomerization domain and leucine-rich repeat-containing X1. This leads to the accumulation of autophagosomes in the cytoplasm and subsequent autophagic cell death [46,47,48,49].

A plethora of downstream targets phosphorylated by ROCK have been implicated in various biological processes that are critical for regulating cell morphology, motility, survival, and apoptosis [7,16]. ROCK activation triggers the phosphorylation of numerous central regulatory proteins, eliciting various cellular responses including autophagy, cell survival, apoptosis, vesicle dynamics, cytoskeletal regulation, cell growth, and regeneration, as well as the modulation of cell morphology and motility [8,11,50]. Several studies have demonstrated the critical function of ROCK activity in programmed cell death, highlighting the significance of the Rho/ROCK signaling pathway in cisplatin-induced apoptosis across human and rodent models [6,8,11,12,13]. After apoptosis initiation, ROCK proteins are activated via caspase-mediated cleavage, subsequently instigating apoptotic membrane blebbing, chromosome condensation, and Golgi body fragmentation through myosin light chain phosphorylation, which are pivotal processes that facilitate final phagocytosis by immune cells [51,52,53]. Furthermore, ROCK activity fosters a feedback loop that perpetuates caspase-3 activation, thereby amplifying cell death [7]. ROCK has been demonstrated to activate the phosphatase and tensin homolog, while inhibiting insulin receptor substrate 1 signaling, leading to the deactivation of protein kinase B (AKT) [6,7,16]. Given the role of AKT in promoting cell survival, its deactivation triggers both extrinsic and intrinsic pathways [7]. Additionally, ROCK is involved in the extrinsic pathway of apoptosis by participating in the clustering of death receptors including the TNF-α and Fas receptors (Figure 4) [7,8].

This study is the first to explore the protective effects of fasudil (a ROCK inhibitor) against cisplatin-induced ototoxicity. Previous studies have shown that fasudil can mitigate neomycin-induced ototoxicity in HEI-OC1 cell lines [18,54]. Fasudil can prevent programmed cell death by inhibiting ROS generation and autophagy, leading to a reduction in ΔΨm induced by neomycin ototoxicity [18,54]. In both studies, the protective effect of fasudil was analyzed by treating hair cells with 5 mM neomycin. When neomycin was administered alone for 24 h, cell viability was about 49–52% compared to the control. However, when co-treated with 20 µM fasudil, cell viability increased to approximately 88%, and with 0.01 µM fasudil, it rose to 79%. In contrast, in the present study, cisplatin alone reduced cell viability to 42.7%, but co-treatment with 100 µM fasudil increased cell viability to 57.3%. Although this study was conducted using zebrafish, it is possible that the protective effect of fasudil may be more pronounced in neomycin ototoxicity than in cisplatin ototoxicity. Another study demonstrated that ORC-13661, a competitive inhibitor of the mechanoelectrical transducer (MET) channels located at the tips of hair cell stereocilia, protects zebrafish lateral line hair cells from cisplatin toxicity [55]. In that study, when 200 µM cisplatin was administered alone for 24 h, hair cell viability decreased to about 19% of the control, but co-treatment with cisplatin and ORC-13661 increased hair cell viability to approximately 74%. Although the concentration and treatment time of cisplatin differ, this comparison suggests that fasudil shows a reduced protective effect against cisplatin ototoxicity than MET channel inhibitors.

In the present study, fasudil exerted a protective effect against hair cell loss in a zebrafish model of cisplatin-induced ototoxicity by suppressing ROS formation; reducing mitochondrial transmembrane potential; and inhibiting caspase-3 activation, autophagy activation, and apoptosis. Given that ROCK participates in both the intrinsic and extrinsic apoptotic cascades induced by cisplatin ototoxicity, it is believed that fasudil inhibits hair cell loss and changes in the rheotactic behavior induced by cisplatin by inhibiting ROCK.

Studies focusing on embryonic development and morphogenesis have analyzed ROCK function in zebrafish models [56,57]. One study demonstrated that the pharmacologically induced inhibition of ROCK resulted in the disruption of germ plasm localization in zebrafish embryos, thereby interfering with microtubular formation [57]. Another study revealed that ROCK suppresses mesoderm induction in zebrafish embryos by regulating TGF-β signaling [56]. Nevertheless, to the best of our knowledge, no research has analyzed the role of ROCK in apoptosis, making this study the first to demonstrate that the inhibition of ROCK in zebrafish hair cells prevents apoptosis induced by cisplatin.

The hair cells within zebrafish neuromasts play a role in shaping various behaviors observed in zebrafish, such as their response to water flow, detecting prey, and avoiding predators [19,58]. Previous studies on zebrafish behavior have shown significant changes in the event of hair cell damage by ototoxic drugs, indicating the potential of zebrafish behavior as a tool for screening ototoxic drugs [23,28,30,39,59]. In this study, we indirectly confirmed hair cell damage by examining rheotaxis, a behavior in zebrafish that is mediated by hair cells [35]. The rheotaxis experiment revealed a significant decrease in zebrafish rheotaxis when hair cell damage was induced by cisplatin ototoxicity, confirming the protective effect of fasudil on rheotaxis [15,30,60,61,62].

ROCK inhibitors have emerged as promising therapeutic agents in both preclinical and clinical settings, targeting diseases such as glaucoma, cardiovascular disorders, neurodegenerative diseases, cancer, and fibrosis [16,63,64,65,66]. In animal models, ROCK inhibitors are administered via oral, intravenous, intraperitoneal, topical, or intracerebral routes to investigate their efficacy in reducing hypertension, promoting neuroprotection, inhibiting tumor metastasis, and alleviating tissue fibrosis [16,64,65,67]. Clinically, ROCK inhibitors like fasudil and ripasudil are used for conditions such as cerebral vasospasm and glaucoma, respectively [66,68]. Despite their broad potential, challenges such as off-target effects, long-term toxicity, and delivery to specific tissues, including the central nervous system, remain. Future advancements in selective inhibition and targeted delivery systems may enhance the therapeutic utility of ROCK inhibitors while minimizing adverse effects, paving the way for their expanded use in treating a variety of human diseases.

ROCK inhibitors have been found to influence not only hair cells but also synaptic ribbons, which play a critical role in auditory signal transmission [17,69]. Synaptic ribbons are specialized presynaptic structures in sensory cells, such as cochlear hair cells, responsible for the rapid and sustained release of neurotransmitters necessary for accurate auditory perception [70,71]. Studies suggest that ROCK signaling regulates synaptic vesicle trafficking and the structural integrity of synaptic ribbons, thereby affecting synaptic efficacy and plasticity [72,73]. In the context of auditory function, aberrations in synaptic ribbon dynamics can lead to impaired auditory signaling and hearing loss [73]. ROCK inhibitors, by modulating these pathways, have shown potential in preserving or restoring synaptic ribbon integrity and function [17,74,75]. These findings highlight the dual role of ROCK inhibitors in protecting hair cells and optimizing synaptic transmission, offering promising therapeutic avenues for addressing hearing impairments [76]. Further research is warranted to elucidate the precise mechanisms by which ROCK inhibitors interact with synaptic ribbons and their long-term impact on auditory health.

This study has a few limitations. First, there was a lack of specific information about the absorption of fasudil into zebrafish hair cells. Additionally, we could not rule out the possibility that fasudil might inhibit the penetration of cisplatin into hair cells. Second, although various types of programmed cell death, such as apoptosis, necroptosis, autophagy, ferroptosis, and pyroptosis, occur during cisplatin ototoxicity, our study focused exclusively on apoptosis. Third, we did not confirm that cisplatin activates the ROCK signaling pathway in the zebrafish model. Fourth, the sample size for the TUNEL, ROS, and TMRE labeling assays, as well as caspase-3 immunostaining, is not large enough. Fifth, as we used a zebrafish model as a screening tool for otoprotective agents, further research is needed before applying this to humans, as there are significant differences in the anatomical structure and cellular composition between the zebrafish lateral line and the mammalian cochlea.

## 4. Materials and Methods

### 4.1. Chemical Preparation

Cisplatin (purity > 99.9% trace metals basis), which was obtained from Sigma Chemical Co. (CAS 15663-27-1, St. Louis, MO, USA), was dissolved in sterile saline (0.9% NaCl) at 37 °C for 12 h under dark conditions. Fasudil hydrochloride (purity > 98%), procured from Tocris Cookson, Ltd. (CAS 105628-07-7, Bristol, UK), was dissolved in distilled water (H_2_O) at 37 °C.

### 4.2. Animal Model

The (Brn3c:EGFP)^s356t^ transgenic zebrafish line expressed enhanced green fluorescent protein (EGFP) in the neuromast hair cells, which facilitates direct hair cell counting [77]. Zebrafish larvae, which were obtained by mating adult zebrafish, were maintained at a temperature of 28.5 °C within the zebrafish facility at Ansan Hospital, Korea University. Embryos were cultured at a density of approximately 50 embryos per 100 mm^2^ of the Petri dish in embryo media (15 mM NaCl, 0.5 mM KCl, 1 mM CaCl_2_, 1 mM MgSO_4_, 0.15 mM KH_2_PO_4_, 0.05 mM NH_2_PO_4_, and 0.7 mM NaHCO_3_) [78,79]. All experiments involving zebrafish larvae were performed after obtaining approval from the Korea University Institutional Animal Care and Use Committee (approval no. KOREA-2022-0097) and adhered to the guidelines outlined by the Animal Care Ethics Committee of the Korea University Medical Center and the National Institute of Health.

### 4.3. Counting Hair Cells in the Neuromasts of Zebrafish Larvae

At 5 days post-fertilization (dpf), the (Brn3c:EGFP)^s356t^ transgenic zebrafish larvae were exposed to either 1 mM cisplatin solution alone or 1 mM cisplatin solution combined with varying concentrations (10, 100, and 500 μM) of fasudil for a duration of 4 h. Each treatment was delivered to groups of 30 larvae each. The concentration and exposure duration of cisplatin were determined empirically based on previous research, utilizing lethal concentration 50% (LC_50_) values required to induce hair cell death in zebrafish, while still preserving their viability [78,79,80,81]. Following exposure to the cisplatin and fasudil solutions, the zebrafish larvae were triple rinsed in embryo media and anesthetized with tricaine (3-aminobenzoic acid 0.4 g in ethyl ester, 100 mL, pH = 7, adjusted with Tris buffer) for 5 min. Subsequently, the larvae were immobilized on a slide using methylcellulose, and the hair cells in the neuromasts of the supraorbital (SO1 and SO2), otic (O1), and occipital (OC1) lateral lines were quantified using a fluorescence microscope (Eclipse Ni-U, Nikon, Japan). These neuromasts were selected because of their simultaneous visibility, eliminating the need to reposition the fish under the microscope. The total hair cell counts in the SO1, SO2, O1, and OC1 neuromasts were calculated for each zebrafish in all experimental and control conditions using a fluorescence microscope (n = 30 per group).

Additionally, to investigate the direct impact of fasudil on hair cells, the hair cell count was compared between the 100 μM fasudil and control groups after a 4 h treatment period. The total hair cell counts in the SO1, SO2, O1, and OC1 neuromasts were measured again under each condition using a fluorescence microscope (n = 20 per group).

### 4.4. Quantitative Assessment of Apoptosis Using Terminal Deoxynucleotidyl Transferase- Mediated dUTP–Biotin Nick End Labeling Assay

Apoptosis in neuromasts was assessed using the terminal deoxynucleotidyl transferase (TdT)-mediated dUTP–biotin nick end labeling (TUNEL) assay with an in situ cell detection kit (12156792910, Roche Molecular Biochemicals, Mannheim, Germany), following the manufacturer’s instructions. The experiment included 5-dpf larvae. The control group (n = 4) was exposed to the embryo media, while the cisplatin group (n = 6) was exposed to 1 mM cisplatin media, and the cisplatin + fasudil group (n = 3) was exposed to media containing 1 mM cisplatin and 100 μM fasudil for 4 h, followed by washing with phosphate-buffered saline and fixation in 4% paraformaldehyde (PC2031-050-00, Biosesang, Gyeonggi-do, Republic of Korea). Subsequently, larvae from each group were treated with 50 μL of the TUNEL reaction mixture (TdT and fluorescein–dUTP) at 37 °C for 60 min in a humid environment. The TUNEL intensity within the four neuromasts (SO1, SO2, O1, and OC1) of the transgenic zebrafish was visualized under fluorescence microscopy (×20; Eclipse Ti2, Nikon, Japan). The TUNEL-positive cells in the four neuromasts were quantified for each zebrafish and the average number was contrasted among the control group, 1 mM cisplatin +100 μM fausdil co-treatment group, and 1 mM cisplatin group [82,83,84].

### 4.5. Quantifying Reactive Oxygen Species Levels in Hair Cells

CellROX™ deep red reagent (C10422, Invitrogen, Carlsbad, CA, USA) was employed to determine the effect of cisplatin and fasudil on reactive oxygen species (ROS) production in zebrafish hair cells. Five-dpf larvae from the control, cisplatin, and cisplatin + fasudil groups were treated with 5 µM CellROX™ deep red in embryo media for 30 min at 28 °C and protected from light (n = 6 per group). Subsequently, the larvae were rinsed with phosphate-buffered saline and fixed in 4% paraformaldehyde. Prior to imaging, the larvae were kept in the dark and the labeling intensity was quantified in the four neuromasts (SO1, SO2, O1, and OC1) using a fluorescence microscope (×20; Eclipse Ti2, Nikon, Japan). The mean ROS intensity of four neuromasts per larva was determined relative to the mean intensity of the control group. Thereafter, the average intensities were compared among the control group, 1 mM cisplatin group, and 1 mM cisplatin +100 μM fasudil co-treatment group [85].

### 4.6. Quantifying Mitochondrial Transmembrane Potential Loss in Hair Cells

We employed positively charged tetramethylrhodamine ethyl ester (TMRE) labeling (T669, Invitrogen, Carlsbad, CA, USA) to assess the loss of mitochondrial membrane potential (ΔΨm) in the hair cells. Five-dpf larvae from the control, cisplatin, and cisplatin + fasudil groups were rinsed three times with embryo media. Subsequently, the larvae were immersed in 250 nM TMRE reagent diluted with embryo media in darkness for 30 min at 28 °C, followed by triple washing and anesthesia with tricaine. The labeling intensity of the larvae was examined in the four neuromasts (SO1, SO2, O1, and OC1) under fluorescence microscopy (Eclipse Ni–U, Nikon, Japan). The average labeling intensity of the four neuromasts per larva was determined relative to the mean intensity of the control group. The average intensities were then compared among the 1 mM cisplatin (n = 4), 1 mM cisplatin + 100 μM fasudil co-treatment (n = 7), and control groups (n = 7) [42,85].

### 4.7. Quantitative Analysis of Caspase 3 Activity

The C8487 Anti-Caspase 3 antibody produced in rabbit (C8487, Sigma-Aldrich, Taufkirchen, Germany) was employed to evaluate caspase 3 activation through proteolytic cleavage in the hair cells. Five-dpf larvae from the control, cisplatin, and cisplatin + fasudil groups were rinsed three times with the embryo media. Subsequently, the larvae were immersed in Anti-Caspase 3 diluted in embryo media for 30 min at 28 °C in darkness, followed by triple washing and anesthesia with tricaine. Thereafter, the intensity was examined in the four neuromasts (SO1, SO2, O1, and OC1) in each group using fluorescence microscopy [86]. The average labeling intensity of the four neuromasts per larva was determined relative to the mean intensity of the control group. Thereafter, the average intensities were compared among the 1 mM cisplatin, 1 mM cisplatin + 100 μM fasudil co-treatment, and control groups (n = 5 per group).

### 4.8. Qualitative Analysis of Autophagy Activation

LysoTracker Red DND-99 (L7528, Invitrogen, Carlsbad, CA, USA) was utilized to assess autophagy activation in the hair cells. Five-dpf larvae from the control, cisplatin, and cisplatin + fasudil groups were washed thrice with embryo media. Subsequently, the larvae were immersed in LysoTracker Red DND-99 diluted in embryo media for 30 min at 28 °C in darkness, followed by triple washing and anesthesia with tricaine. The labeling intensity of the four neuromasts (SO1, SO2, O1, and OC1) in each group of larvae was analyzed using fluorescence microscopy [87].

### 4.9. Quantification of Hair Cell Viability

FM1-43FX (F35355, Invitrogen, Carlsbad, CA, USA) vital dye labeling was used to confirm mechanotransduction in the surviving hair cells. Five-dpf larvae from the control, cisplatin, and cisplatin+ fasudil groups were washed thrice with embryo media (n = 6 per group). Subsequently, the larvae were incubated in 3 μM FM1–43FX solution in darkness for 60 s, followed by triple washing and anesthesia with tricaine. The labeling intensity in four larval neuromasts (SO1, SO2, O1, and OC1) was observed using fluorescence microscopy. The mean FM1-43FX intensity of the four neuromasts of each larva was determined relative to that of the control group. Subsequently, the average labeling intensities relative to the control group were compared among the other experimental groups [41,88].

### 4.10. Rheotaxis Assessment

**Figure 5:**
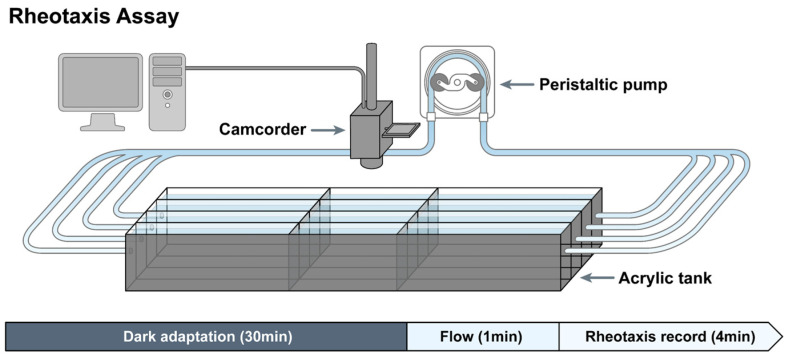
Rheotaxis assay setup. The apparatus consists of three lanes with continuous flow regulated by an attached pump. An overhead camera simultaneously captures the behaviors of all the larvae within each lane.

To investigate rheotaxis, a specialized acrylic tank with three separate lanes was constructed. Each lane measured 3 cm in height, 2 cm in width, and 61 cm in length, featuring two stainless steel screens positioned midway to delineate a 10-cm-long observation area. The lanes were filled with embryo medium to a depth of 2.8 cm, and a peristaltic pump (WPI Peri-StarTM PRO) was employed to sustain a continuous laminar flow at a rate of 0.5 cm/s. A previous study conducted in our laboratory validated the velocity and parabolic nature of laminar flow by introducing blue ink [29]. The recirculating medium’s temperature was maintained at 28.5 °C. The experiments were conducted in a darkened environment to minimize the impact of visual stimuli on zebrafish behavior. Their movements were captured using a camcorder (SONY, FDR-AX700, Japan) with infrared illumination and were recorded at 30 frames per second (fps). Five-dpf zebrafish larvae were exposed to either 1 mM cisplatin or a combination of 100 μM fasudil and 1 mM cisplatin for 4 h. Ten larvae from each experimental group, including the cisplatin only, cisplatin + fasudil, and control groups, were placed within the observation zone of each lane. After allowing the larvae to acclimate to the no-flow condition for 30 min, medium flow was initiated within the lanes at 0.5 cm/s for 1 min, followed by a 4 min observation period to analyze larval behavior. Still images were extracted from the recordings every 2.5 s to assess the absolute angles of rheotaxis exhibited by the larvae. The absolute angle of rheotaxis was measured as the angle formed by the body of the larva in the direction opposite to water flow [31]. Each group consisted of 10 larvae, and the experiment was repeated four times using different larvae under identical conditions. The absolute angles of rheotaxis exhibited by the larvae in each still image were measured, where the larvae oriented themselves in the direction opposite to the flow of water, and compared across groups (Figure 5, Movie S1).

### 4.11. Statistical Analysis

We conducted statistical analyses for multiple groups using appropriate methods, viz. the one-way analysis of variance (ANOVA) for normally distributed variables and the Kruskal–Wallis test for variables with non-normal distribution. Tukey’s honest significant difference test and Bonferroni correction were employed for post hoc analyses, as appropriate. Statistical significance between two groups with non-normal distribution was assessed using the Mann–Whitney U test. *p*-values <0.05 were considered statistically significant (*** *p* < 0.001, ** *p* < 0.01, * *p* < 0.05). All statistical analyses were performed using SPSS v22.0 and all data were presented as means ± standard errors.

## 5. Conclusions

This study demonstrates that cisplatin induces apoptosis, resulting in hair cell loss in zebrafish larvae. Fasudil, a ROCK inhibitor, apparently possesses the ability to safeguard zebrafish hair cells against cisplatin ototoxicity and inhibit apoptosis. Nevertheless, additional research is required to clarify the mechanisms behind these protective effects and to assess the translational prospects of fasudil in preventing cisplatin-induced hearing loss in clinical settings.

## Figures and Tables

**Table 1:** Average number of hair cells.

Group	SO1	SO2	O1	OC1	Sum (%)
Control (n = 30)	9.8 ± 1.4	11.0 ± 2.2	10.7 ± 1.5	11.6 ± 2.4	43.1 ± 3.6 (100%)
Cisplatin 1 mM (n = 30)	5.3 ± 1.5	4.2 ± 2.3	4.0 ± 2.3	5.0 ± 3.2	18.4 ± 7.9 (43%)
Cisplatin 1 mM + Fasudil 10 μM (n = 30)	5.8 ± 1.9	5.7 ± 2.4	5.4 ± 2.5	6.0 ± 3.4	22.9 ± 8.2 (53%)
Cisplatin 1 mM + Fasudil 100 μM (n = 30)	5.9 ± 1.6	6.0 ± 2.4	6.4 ± 2.2	6.4 ± 2.7	24.7 ± 6.7 (57%)
Cisplatin 1 mM + Fasudil 500 μM (n = 30)	5.7 ± 1.9	6.1 ± 2.6	6.9 ± 1.8	6.7 ± 6.3	25.5 ± 6.2 (59%)

Cisplatin induced a marked loss in the number of hair cells within the neuromasts, whereas fasudil conferred considerable protection against cisplatin-induced hair cell loss at concentrations of 100 μM and 500 μM.

## Data Availability

The datasets generated and/or analyzed in the current study are available from the corresponding author upon reasonable request.

## Supplementary Materials

The following supporting information can be downloaded at: https://www.mdpi.com/article/10.3390/ijms252413363/s1.
